# Evaluation of extracorporeal shock wave therapy for refractory angina pectoris with quantitative analysis using cardiac magnetic resonance imaging: a short communication

**DOI:** 10.1007/s12471-016-0825-7

**Published:** 2016-04-08

**Authors:** J. Slikkerveer, K. de Boer, L. F. H. J. Robbers, A. C. van Rossum, O. Kamp

**Affiliations:** Department of Cardiology and Institute of Cardiovascular Research, VU University Medical Center, De Boelelaan 1117, 1081 HV Amsterdam, the Netherlands; Interuniversity Cardiology Institute of the Netherlands, Utrecht, the Netherlands

**Keywords:** Shockwave therapy, Quantitative analysis, Myocardial perfusion, Refractory angina pectoris, Cardiac MRI

## Abstract

**Aims:**

There is a continuing search for new treatment options in patients who suffer from refractory angina pectoris to improve quality of life. Several studies have recently demonstrated promising results by stimulating angiogenesis using extracorporeal shockwave therapy in these patients. The purpose of this study is to quantitatively analyse the effect of extracorporeal shockwave therapy on myocardial perfusion in patients with refractory angina pectoris.

**Methods:**

We included 15 patients with NYHA class 3–4 of whom 8 patients underwent baseline and follow-up cardiac magnetic resonance imaging (CMR). All patients received 9 shockwave treatments of their ischaemic zone over a period of 3 months.

**Results:**

Quantitative analysis of myocardial perfusion using CMR revealed no significant improvement of myocardial perfusion after treatment (0.80 ± 0.22 vs 0.76 ± 0.31; *p* = 0.42). However, the total group of 15 patients did experience a significant improvement in NYHA class (*p* = 0.034) and reduction of nitroglycerin use (*p* = 0.012).

**Conclusion:**

Although treatment with extracorporeal shockwave was associated with an improvement in NYHA class, we could not observe an improvement in myocardial ischaemic zone and perfusion with CMR. To unravel the exact mechanisms of shockwave treatment, more *in vitro *and animal studies as well as larger (placebo-controlled) studies are required.

## Introduction

Despite maximal pharmacotherapy and revascularisation procedures there is a growing number of patients who suffer from refractory angina pectoris, with an estimated incidence between 5 and 10 % and a 9-year mortality of almost 30 % [1, 2]. As a result, there is a continuing search for new treatment options for this specific patient group. Several therapeutic interventions have been tried to improve quality of life (thoracic epidural analgesia [3], transcutaneous electrical nerve stimulation [4], spinal cord stimulation [5]) and/or reduce myocardial ischaemia (enhanced external counterpulsation [6], percutaneous transmyocardial laser revascularisation [7], and gene therapy [8]).

Recently, a new noninvasive treatment strategy stimulating angiogenesis has gained attention [9–11]. Extracorporeal shockwave therapy uses high-intensity acoustic pulsed waves to stimulate angiogenesis. Based on a pre-clinical study the intensity used is approximately 10 % of the intensity used with lithotripsy [9]. In an *in vivo* pig model with chronic occlusion of the left circumflex coronary artery, local application of shockwave therapy improved left ventricular ejection fraction, wall thickening fraction and myocardial blood flow in the ischaemic region after 4 weeks compared with non-treated pigs [9]. Furthermore, histology also showed an increase in capillary density in the treated pigs as well as an upregulation of vascular endothelial growth factor (VEGF) mRNA expression.

The first small studies in humans show promising results based on the Canadian Cardiovascular Society class, exercise testing and qualitative analysis of SPECT images [11–15]. However, no quantitative analysis has been performed to objectively measure the myocardial blood flow in the treated ischaemic zone.

Therefore, we initiated this study to quantitatively examine the effect of shockwave therapy on the ischaemic zone in patients with refractory angina pectoris. We hypothesise that shockwave therapy may improve New York Heart Association (NYHA) class by decreasing the size of the ischaemic zone and increasing myocardial blood flow in patients with refractory angina pectoris using cardiac MRI (CMR).

## Methods

### Patient selection

Patients of 18 years or older suffering from refractory angina pectoris where included in the study. Refractory angina pectoris is defined as having angina pectoris NYHA class 3–4 based on documented ischaemia without any possible revascularisation option left, either PCI or CABG, under a maximum tolerable dose of medication. Patients were excluded from the study when they suffered from severe heart valve disease, intraventricular thrombus, a malignancy in the area of treatment, chronic lung disease (including emphysema and pulmonary fibrosis), a myocardial infarction in the 3 months prior to the start of the study, had a contraindication for undergoing CMR, or when the patient was pregnant.

The protocol was approved by the medical ethics committee of the VU University Medical Center and all patients provided written informed consent.

### CMR

After refraining from intake of competitive antagonists of adenosine in the 24 hours prior to examination, CMR was performed on a clinical 1.5 Tesla scanner (Avanto, Siemens, Erlangen, Germany) using a phased-array cardiac receiver coil. All images were ECG gated and acquired during mid-expiratory breath-holds of 10 to 15 seconds, depending on the heart rate.

Using the four-chamber long-axis cine in the end-systolic phase, 3 short-axis slice positions were determined for myocardial perfusion imaging, at 25, 50 and 75 % of the distance between the mitral valve annulus and the apex. Prior to imaging, patients received intravenous adenosine at a rate of 140 µg/kg/min for 3 minutes. Image acquisition was initiated simultaneously with the administration of a 0.1 mmol/kg bolus of a gadolinium-based contrast agent (Magnevist, Schering AG, Berlin, Germany) at a rate of 3 ml/sec. Images were acquired at the 3 aforementioned levels during each cardiac cycle, for a total duration of 50 cardiac cycles. Adenosine infusion was discontinued immediately after the images were obtained.

**Table 1:** Sequence parameters for myocardial perfusion with CMR

Sequence – Parameter	
*Steady-state free precession cine imaging*	
Spatial resolution (frequency encoding dir.)	1.3–1.6 mm
Spatial resolution (phase encoding dir.)	1.8–2.2 mm
Slice thickness	5.0 mm
Slice gap	5 mm
Flip angle	75°
Field-of-view size	360–400 mm
Matrix size	256 × 256
Percentage phase field of view	80–90 %
Echo time	1.54 ms
Temporal resolution	34–38 ms
*First-pass perfusion – single-shot saturation recovery gradient echo*	
Spatial resolution (frequency encoding dir.)	2.2–2.5 mm
Spatial resolution (phase encoding dir.)	2.2–2.5 mm
Slice thickness	8.0 mm
Slice gap	10–17 mm
Field-of-view size	360–400 mm
Matrix size	160 × 160
Percentage phase field of view	100 %
Time of repetition	154.8 ms
Echo time	1.0 ms
Flip angle	18°
Acceleration technique	Echo planar imaging
Duration	50 cardiac cycles
*T1-weighted inversion recovery gradient echo*	
Spatial resolution (frequency encoding dir.)	1.3–1.6 mm
Spatial resolution (phase encoding dir.)	1.6–1.9 mm
Slice thickness	5.0 mm
Slice gap	5.0 mm
Flip angle	25°
Field-of-view matrix	256 × 256
Percentage phase field of view	80–95 %
Time of repetition	1 x RR interval
Echo time	4.4 ms
Inversion time	250–400 ms

At least 10 minutes after stopping adenosine infusion and myocardial perfusion acquisition, resting myocardial perfusion imaging was performed, using the same sequence parameters and contrast dose (total cumulative dose 0.2 mmol/kg). The sequence parameters used for cine and myocardial perfusion imaging are mentioned in Table 1.

### Shockwave treatment

Shockwave therapy was applied using the Cardiospec (Medispec, Germantown, Maryland). The location of the ischaemic zone was detected by consensus of 2 experienced observers based on visual inspection of CMR, using regional function. This area was translated into the 16-segment model of echo and divided into three parts. In order to make sure we covered the entire ischaemic zone we enlarged the treatment area. Therefore, more regions were treated than located on CMR. The treatment protocol covered a total period of 9 weeks in which patients were treated in weeks 1, 5 and 9. During each treatment week the patient visited the outpatient clinic 3 times, resulting in a total of 9 treatments.

Patients were positioned in a supine position and connected to continuous ECG monitoring. An S3 ultrasound probe, which was linked to a Sonos 4500 (Philips, Best, the Netherlands) and connected to the Cardiospec, was positioned on the chest of the patient so that the ischaemic treatment zone was located in the centre of the ultrasound image. The depth of the ischaemic zone was measured using a caliper on the ultrasound machine. Based on these measurements a balloon with a shockwave electrode inside and attached to the Cardiospec was placed on a patient’s chest at the appropriate position. Using an ellipsoid reflector inside the balloon enabled us to focus the shockwave with the right intensity to any possible depth. The intensity used was based on pre-clinical studies where an optimal dosage-effect relation was shown at approximately 10 % of the intensity used with lithotripsy, as was mentioned earlier [9]. Since the depth and location of the machine could be adjusted we were able to apply the same amount of energy to two remote distinct treatment zones. The balloon was inflated with saline water (5 % NaCl) until a good connection with the skin was achieved. Prior to inflation of the balloon a large amount of gel was applied to the skin in order to prevent any air becoming trapped between the skin and the balloon. Once installed, a total of 100 shocks per spot were applied. The amount of spots treated during a session depended on the size of the ischaemic zone, with every spot covering an area of approximately 1 by 1 cm.

### Analysis and definitions

Analysis was performed off-line using dedicated software (MASS v.5.1 2010-EXP beta, Medis, Leiden, the Netherlands) by consensus of 2 experienced observers. All analyses were performed on short-axis images with a 16-segmental distribution of the myocardium. Using the cine images, myocardial volumes during the end-diastolic and end-systolic phase were calculated. From the volumes, stroke volume and ejection fraction were calculated. The location of the treatment zone was based on visual inspection by consensus of 2 experienced observers.

**Fig. 1:**
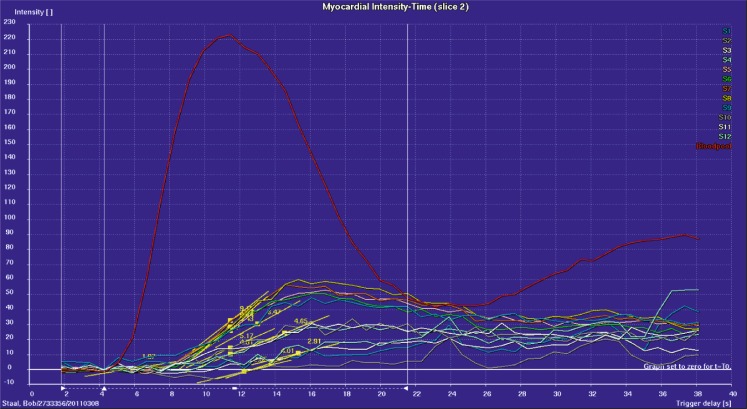
Myocardial perfusion of all 12 segments per region (base, mid or apex) by calculation of the maximal relative upslope (yellow lines), corrected for baseline signal intensity and the arterial input by the upslope of the blood pool curve (red line); a steeper yellow line means a better myocardial perfusion

Myocardial perfusion was evaluated quantitatively by calculation of the maximal relative upslope [%], using the maximum upslope [au·s^−1^] of the myocardial signal-intensity-versus-time curves and by correcting for baseline signal intensity [au] and the arterial input by the upslope of the blood pool curve [au·s^−1^] (Fig. 1). As mentioned above, in order to make sure we treated the entire ischaemic zone we enlarged the treatment area, resulting in treatment of healthy segments. To reduce the effect of these treated healthy segments and to reduce the effect of artifacts on analysis we divided the 3 short-axis views (base, mid and apical) into 12 segments per axis. Previous studies have already proven the relative upslope to be an accurate and reproducible parameter for quantitative perfusion analysis [16–18].

### Questionnaire

Prior to the start of shockwave treatment all patients were asked to answer the Seattle Angina questionnaire to estimate NYHA class, the use of nitroglycerin and the frequency of angina pectoris. The same questionnaire was used 3 months after final treatment during follow-up CMR.

### Statistical analysis

Categorical data are presented as frequencies (percentage), and continuous data as mean ± SD. Comparisons between means of groups were performed using Student’s T‑test (2 groups) and the Wilcoxon signed-rank test in case of ordinal variables. Obtained data were analysed using SPSS 21.0 (Chicago, USA).

## Results

Between March 2009 and November 2011, a total of 15 patients underwent shockwave therapy, of whom 8 underwent serial baseline and follow-up CMR. Due to claustrophobia (*n* = 1), obesity (*n* = 1) or an implantable device (*n* = 5), the remaining 7 patients were not analysed using CMR. Of these 8 patients, 6 had a CABG and 1 or more PCIs in their medical history and 2 only PCIs. All patients had 1 or more chronic total occlusion of their native system at inclusion. In 3 of these patients all 3 vessels of the native system had a chronic total occlusion and in 2 out of 8 patients there were 2 chronic total occluded vessels. Interestingly, only 2 of these 8 patients had collateral flow on their coronary angiogram. All patients underwent a total of 9 shockwave treatments, without any adverse events. No arrhythmias were noticed during treatment. The most encountered complaint after treatment was tiredness, which lasted for a maximum of 24 hours. Furthermore, all patients returned their questionnaires at baseline and follow-up.

### CMR

Baseline and follow-up results are mentioned in Table 2. There was no significant difference in left ventricular volumes and ejection fraction between baseline and follow-up results. We did not find a significant difference in the myocardial perfusion ratio index, which is the relative ratio of the maximum upslope of stress to rest normalised to left ventricle input: 0.79 ± 0.11 vs 0.72 ± 0.24 (*p* = 0.40). For analysis of the myocardial perfusion ratio index the left ventricle was divided into a base, mid and apical part during analysis using CMR and each part contained 12 segments, in total 36 segments per patient. In one patient the left ventricular base images during follow-up were not suitable for analysis. Therefore, a total of 276 segments out of the initially available 288 segments (= 96 %) were analysed. Based on visual inspection, a total of 77 segments were marked as ischaemic segments and received treatment. Also, when group comparison was performed between the 77 treated and 199 untreated segments based on the myocardial perfusion ratio index there was no significant difference: 0.80 ± 0.22 vs 0.76 ± 0.31 (*p* = 0.42).

**Table 2:** Statistical analysis of CMR characteristics (*n* = 8)

	Baseline	Follow-up	*p*-value
EDV	164.4 ± 49.2	168.5 ± 54.4	0.62
ESV	81.9 ± 40.2	86 ± 41.4	0.36
EF	51.8 ± 15.2	51.5 ± 16.5	0.89
MPRi treated zones	0.80 ± 0.22	0.76 ± 0.31	0.42
MPRi total	0.79 ± 0.11	0.72 ± 0.24	0.40

*EDV* end-diastolic volume, *ESV* end-systolic volume, *EF* ejection fraction, *MPRi* myocardial perfusion ratio index (relative upslope of stress to rest, normalised to LV input)

### Secondary endpoints

Neither NYHA class or frequency of nitroglycerin showed a significant improvement in patients analysed with CMR (*p* = 0.102 and* p* = 0.480, respectively). Although, a trend to NYHA class improvement was observed after treatment.

**Fig. 2:**
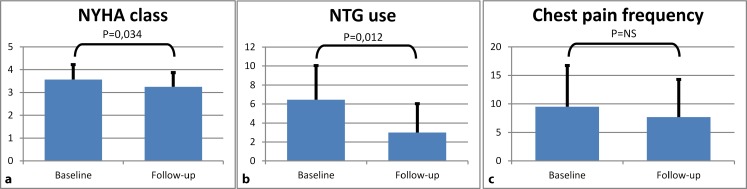
Effect of shockwave therapy on NYHA class (**a**), nitroglycerin use (**b**), and chest pain frequency (**c**). Y‑axis shows frequency per week for nitroglycerin use and chest pain frequency. Results are shown as mean ± SD

When the total group of 15 patients was analysed, as shown in Fig. 2, we found a significant improvement in NYHA class and reduction in nitroglycerin use (*p* = 0.034 and *p* = 0.012 respectively). Frequency of chest pain did not show a significant improvement (*p* = 0.405).

## Discussion

To our knowledge, this is the first quantitative study examining the effect of shockwave therapy on myocardial ischaemia and perfusion. As has been shown previously, our patients also experienced an improvement in NYHA class and reduction in nitroglycerin use (Fig. 2). Nevertheless, in contrast to earlier studies, using qualitative analysis, we could not demonstrate an improvement in myocardial perfusion. Therefore, NYHA class improvement and reduction in nitroglycerin use could not be explained solely by improvement of myocardial perfusion after treatment [11–14].

Table 3 summarises the studies performed thus far using shockwave therapy to treat refractory angina pectoris patients. One can observe the diverse treatment strategies used and difference in time to analysis. In the study performed by Fukumoto et al., patients underwent further treatment based on SPECT, resulting in severe bias [11]. Vasyuk et al., Yang et al. and Prasad et al. had similar study protocols to ours. In contrast to our results, they did demonstrate improvement in perfusion after different follow-up periods, either early (1 month) or late (3–9 months) after completion of shockwave therapy [13–15]. This might be explained by the fact that we used CMR in contrast to others who used SPECT, where CMR has been demonstrated in the past to be superior regarding perfusion measurement in myocardial ischaemia in general and distinguishing between epicardial and endocardial perfusion in particular, compared with SPECT, due to its higher spatial resolution [19]. Furthermore, it also reliably identifies perfusion abnormalities when compared with PET and coronary angiography [20]. A placebo-controlled trial performed with more frequent CMR analysis during follow-up would overcome this issue in future studies. The difference in results of our and earlier performed studies also reveals a potential lack in our knowledge of the exact working mechanism of shockwave therapy.

**Table 3:** Overview of studies performed with shockwave therapy and use of treatment schedule and time to analysis

Authors	Treatment schedule	Time to analysis	Results
Fukumoto et al. [11]	Based on SPECT results	1, 3, 6 and 12 months after final treatment	Improvement in myocardial perfusion, CCS class and reduction of NTG use, lasting 12 months
Schmid et al. [12]	9 treatments in 3 months	3 months after final treatment	Improvement in symptoms
Vasyuk et al. [13]	9 treatments in 3 months	6 months after final treatment	Improvement in symptoms, myocardial perfusion and LVEF
Yang et al. [14]	9 treatments in 3 months	1 month after final treatment	Improvement in symptoms, myocardial perfusion and LVEF
Prasad et al. [15]	9 treatments in 3 months	3 and 6 months after final treatment	Improvement in symptoms and myocardial perfusion

*CCS* Canadian Cardiovascular Society; *NTG* nitroglycerin; *LVEF* left ventricular ejection fraction

Nishida et al. studied the effect of extracorporeal shockwave in an *in vivo* pig model [9]. Using real-time polymerase chain reaction and Western blotting they demonstrated an increased upregulation of VEGF in pigs receiving treatment. Furthermore, they showed an increase in visible coronary arteries, capillary density (using factor VIII staining), and regional myocardial blood flow. As the authors mention, the exact mechanism remains to be elucidated. Furthermore, it is unknown whether a chronic myocardial ischaemia model of 4 weeks in an otherwise healthy pig can be compared with chronic ischaemia of several months to years in our patients with multiple comorbidities, which might have an effect on biological mechanisms.

Another important issue to take into consideration is treatment of viable tissue, which was shown in another study from the same group [10]. They demonstrated an effect of shockwave on further improvement of left ventricular remodelling after acute myocardial infarction. However, this effect was only observed in patients treated shortly after myocardial infarction. As they did not observe a significant effect when treatment was started several weeks after myocardial infarction, this stresses the importance of viable tissue for shockwave therapy to create an effect. To ensure that we treated viable, ischaemic tissue 2 experienced CMR observers located the ischaemic zones.

Evidently, our study is limited by the small sample size and the lack of a placebo control group. Furthermore, exact quantification of the ischaemic zone using MRI proved difficult, since part of the treated zone contained healthy segments in order to be sure the entire ischaemic zone received treatment. Therefore, we divided each plane into 12 segments to perform the quantification and minimise the effect of artifacts and large segments which received minimal treatment. Consequently, possible damage of these treated healthy segments cannot be excluded. Although we obtained good quality images in all patients, we did experience motion artifacts due to breathing during perfusion imaging, which might result in ghosting and therefore influence our measurements [21]. To acquire the best therapeutic strategy using shockwave in patients with refractory angina pectoris, a better understanding of the exact working mechanism plays a pivotal role. We would therefore suggest going back to bench to perform *in vitro* and animal studies to elucidate the exact working mechanism, thereby revealing which patients may benefit from this treatment option, prior to performing placebo-controlled trials.

In conclusion, our study demonstrates that shockwave therapy improved NYHA functional class and reduced nitroglycerin use in our patient group. However, as we did not observe any improvement in CMR-measured myocardial perfusion and subsequent ischaemic burden, the responsible mechanisms need to be elucidated.
